# ClaR—a novel key regulator of cellobiose and lactose metabolism in *Lactococcus lactis* IL1403

**DOI:** 10.1007/s00253-014-6067-y

**Published:** 2014-09-20

**Authors:** Tamara Aleksandrzak-Piekarczyk, Lidia Stasiak-Różańska, Jarosław Cieśla, Jacek Bardowski

**Affiliations:** 1 Institute of Biochemistry and Biophysics, Polish Academy of Sciences (IBB PAS), Pawińskiego 5a, 02-106 Warsaw, Poland; 2 Department of Biotechnology, Microbiology and Food Evaluation, Faculty of Food Sciences, Warsaw University of Life Sciences, Nowoursynowska 159c, 02-776 Warsaw, Poland

**Keywords:** Lactose assimilation, Cellobiose assimilation, Phosphotransferase system (PTS), Genes regulation, *Lactococcus lactis*

## Abstract

In a number of previous studies, our group has discovered an alternative pathway for lactose utilization in *Lactococcus lactis* that, in addition to a sugar-hydrolyzing enzyme with both P-β-glucosidase and P-β-galactosidase activity (BglS), engages chromosomally encoded components of cellobiose-specific PTS (PTS^Cel-Lac^), including PtcA, PtcB, and CelB. In this report, we show that this system undergoes regulation via ClaR, a novel activator protein from the RpiR family of transcriptional regulators. Although RpiR proteins are widely distributed among lactic acid bacteria, their roles have yet to be confirmed by functional assays. Here, we show that ClaR activity depends on intracellular cellobiose-6-phosphate availability, while other sugars such as glucose or galactose have no influence on it. We also show that ClaR is crucial for activation of the *bglS* and *celB* expression in the presence of cellobiose, with some limited effects on *ptcA* and *ptcB* activation. Among 190 of carbon sources tested, the deletion of *claR* reduces *L. lactis* growth only in lactose- and/or cellobiose-containing media, suggesting a narrow specificity of this regulator within the context of sugar metabolism.

## Introduction

Lactic acid bacteria (LAB), including *Lactococcus lactis*, are the focus of intensive research within the field of carbohydrate catabolism and its regulation, important during industrial fermentation processes (Mayo et al. 2010). The most prevalent pathway for carbohydrate uptake in LAB is the phosphoenolpyruvate-phosphotransferase system (PEP-PTS), which links sugar transport with phosphorylation of incoming sugars. Phosphoenolpyruvate serves as the first phosphoryl donor to Enzyme I, which utilizes a complex phosphoryltransfer cascade to phosphorylate sugars entering bacteria via the transmembrane protein—EIIC (Lorca et al. 2010). Among a wide array of carbohydrates that can be internalized via PTS, in LAB these systems are best known for efficient transport of milk (lactose) and plant (β-glucosides) sugars, which are highly important for industrial fermentation processes involving LAB species.

Due to the economic importance of lactose fermentation, the metabolism of this sugar is studied extensively in *L. lactis*. These studies mostly focus on two pathways: (i) plasmid-localized lactose-specific PTS (PTS^Lac^) and (ii) the chromosomally encoded lactose permease-β-galactosidase system (Aleksandrzak-Piekarczyk 2013). The latter system transports lactose in an unphosphorylated form that enables its subsequent cleavage by β-galactosidase. Recently, we reported on the discovery of a third, alternative lactose uptake system that we found to be operative in *L. lactis* IL1403 (Aleksandrzak-Piekarczyk et al. 2011). This novel lactose utilization pathway engages chromosomally encoded components of a cellobiose-specific PTS (PTS^Cel-Lac^). The proteins of PTS^Cel-Lac^ are encoded in two distinct regions that encompass *ptcA*, *ptcB* (one region), and *celB* (second region), which code for EIIA, EIIB, and EIIC, respectively (Aleksandrzak-Piekarczyk et al. 2011). The latter region also contains the *bglS* gene, which encodes a P-sugar-hydrolyzing enzyme with both P-β-glucosidase and P-β-galactosidase activity. This BglS enzyme has been shown to promote the cleavage of cellobiose and lactose internalized via PTS^Cel-Lac^ in *L. lactis* IL1403 (Aleksandrzak-Piekarczyk et al. 2005). Therefore, PtcAB, CelB, and BglS form a complete system specific for cellobiose and lactose uptake and hydrolysis in *L. lactis* IL1403. We surmise that the existence of PTS^Cel-Lac^ is not limited to *L. lactis* IL1403 and may occur commonly among other lactococcal strains (Aleksandrzak-Piekarczyk et al. 2011). This was confirmed by the later study of Solopova et al. (2012) in *L. lactis* MG1363. In this bacterium, the alternative lactose metabolism pathway, despite being differently induced (by lactose or cellobiose), relies on the same PTS^Cel-Lac^ components used by *L. lactis* IL1403.

The efficiency of transport and subsequent metabolism of incoming sugars is tightly controlled by several regulatory proteins, which form the regulatory network necessary for sensing environmental conditions and setting catabolic capacities of the cell. Guédon et al. (2002) distinguish two groups of regulators—general and secondary regulators. The main example of such a general regulator is catabolite control protein A (CcpA) (Hueck and Hillen 1995). CcpA is a well-conserved protein operating in many low-GC Gram-positive bacteria and, together with its corepressor Ser-P-HPr, it binds to 14-nucleotide *cis*-acting DNA target sites known as catabolite responsive elements (*cre*), promoting carbon catabolite activation (CCA) or repression (CCR) (Weickert and Chambliss 1990). In *L. lactis* strains, CcpA has been shown to repress different genes associated with uptake of β-glucosides, fructose, galactose, or lactose, while activating the glycolytic *las* operon (Aleksandrzak-Piekarczyk et al. 2005, 2011; Barrière et al. 2005; Luesink et al. 1998; Monedero et al. 2001).

In addition to global regulators, carbon catabolism might also be controlled by specific and local secondary regulators belonging to different protein families such as LacI, LysR, AraC, GntR, DeoR, RpiR, or BglG, which are widely distributed among LAB. Regulators from some of these families have been shown to control genes encoding utilization of α-galactosides, β-glucosides, fructose, lactose, maltose, sorbose, sucrose, and xylose in lactococci (Bardowski et al. 1994; Boucher et al. 2003; Mayo et al. 2010).

The YebF protein (now designated cellobiose-lactose regulatory protein—ClaR) from *L. lactis* IL1403, belongs to the RpiR family of regulators. In bacterial genomes, there are many genes that encode potential regulators belonging to the RpiR family (http://www.genome.jp/kegg/ssdb/). However, none of these genes have thus far been shown to play a role in carbohydrate catabolism among LAB. We previously suggested the probable involvement of ClaR in the activation of *bglS*, *celB*, *ptcA*, and *ptcB* genes, which encode components of the novel and the only pathway for lactose utilization in *L. lactis* IL1403 (Aleksandrzak-Piekarczyk et al. 2005, 2011). It has been demonstrated that the expression of these genes is tightly regulated by the general catabolite repression system, whereas *bglS* and *celB* also require the presence of cellobiose to be fully induced (Aleksandrzak-Piekarczyk et al. 2011).

In this report, we show that in *L. lactis* IL1403, genes encoding the PTS^Cel-Lac^ components in the presence of cellobiose are regulated via a novel activator ClaR. Moreover, global phenotypic analysis via phenotype microarray suggests that ClaR is a specific regulator indispensable for cellobiose and lactose metabolism.

## Materials and methods

### Bacterial strains, media, and plasmids

Bacterial strains and plasmids used in this study are listed in Table 1. *Escherichia coli* cells were grown in Luria-Bertani (LB) medium (Wood 1983) at 37 °C, while *L. lactis* was cultivated in M17 glucose medium (G-M17) (Terzaghi and Sandine 1975) or in chemically defined medium (CDM) (Sissler et al. 1999) supplemented with 1 % glucose (G-CDM), 1 % cellobiose (C-CDM), 1 % lactose (L-CDM), 1 % galactose (Gal-CDM), 1 % arbutin (A-CDM), 1 % galactose with 1 % cellobiose (GalC-CDM), or 1 % lactose together with an inducing (0.01 %) concentration of cellobiose (LC-CDM). When required, ampicillin (Amp; 100 μg ml^−1^ for *E. coli*) or erythromycin (Em; 100 μg ml^−1^ for *E. coli* and 5 μg ml^−1^ for *L. lactis*) were added to the medium. Solidified media contained 1.5 % agar and, when necessary, IPTG (isopropyl β-d-thiogalactopyranoside; 1 mM for *E. coli*) and X-gal (5-bromo-4-chloro-3-indodyl-β-d-galactopyranoside; 50 μg ml^−1^ for *E. coli*).

**Table 1:** Bacterial strains, plasmids, and primers

Strain^a^, plasmid, or primers pair	Relevant genotypic or phenotypic properties	Source and/or reference
Strains		
*L. lactis*		
IL1403	Lac^−^, Cel^+^, plasmid-free wild-type, host strain	INRA; Chopin et al. (1984)
LL302	*L. lactis* MG1363-derivative, RepA^+^	Leenhouts et al. (1998)
IL1403Δ*claR*	Lac^−^, Cel^−^, Δ*claR*, Em^s^, plasmid-free, IL1403-derivative	This study
IL1403Δ*ccpA*	Lac^+^, CcpA^−^ (IS*S1*), Em^s^, plasmid-free, IL1403-derivative	Aleksandrzak-Piekarczyk et al. (2005)
IL1403Δ*ccpA*Δ*claR*	Lac^−^, Cel^−^, CcpA^−^ (IS*S1*), Δ*claR*, Em^s^, plasmid-free, IL1403-derivative	This study
IL1403Δ*claR*pIL253*claR*	Lac^−^, Cel^+^, Em^r^, IL1403Δ*claR*-derivative carrying pIL253*claR*	This study
*E. coli*		
TG1	Δ(*hsdMS-mcrB*) 5 Δ(*lac-proAB*) *supE thi-1* F’(*traD36 proAB* ^+^ *lacI* ^q^ *Z*Δ*M15*)	Laboratory collection
Plasmids		
pGEM-T	Amp^r^, M13*ori*, linear T-overhang vector	Promega
pGhost9	Em^r^, *repA* (Ts)	INRA; Maguin et al. (1996)
pGBT58	Km^r^, 10.35 kb, pSC101 replicon, carrying *xylE* under *trfAp*	Jagura-Burdzy et al. (1992)
pIL253	Em^r^, high-copy number lactococcal vector	Simon and Chopin (1988)
pJIM2374	Em^r^, integrative vector carrying *luxAB* genes	Delorme et al. (1999)
Recombinant plasmids		
pJIM2374*claRp*	Em^r^, integrative vector carrying *luxAB* under the control of the putative *claR* promoter (*claRp*)	This study
pGBT58*yebEt*	Km^r^, pGBT58 derivative, carrying the *yebE* terminator (*yebEt*) between *trfAp* and *xylA*	This study
pGhost9Δ*claR*	Em^r^, pGhost9 derivative, carrying the *claR* upstream and downstream DNA regions	This study
pIL253*claR*	Em^r^, pIL253 vector carrying the *claR* gene under the control of its promoter	This study
Primers^b^		
For cloning of *yebE* promoter and terminator or deletion and complementation of the *claR* gene		
*claR*PstI/*claR*ERpr	AA**CTGCAG**GGGTGTTACATTCCAGC/G**GAATTC**GCTCCGCCACAAATTC	
*claR*ERrv/*claR*SalI	G**GAATT**CGCAACTCCCAGTACTAGC/ACGC**GTCGAC**GCAGGCGTTGTTCTTGACC	
yebEfor/*claR*rev	GCTTTTGTTGGTCTGATT/CGGATACTGTTTGGACC	
*claR*Smaf/*claR*PstR	CCCGGGCTTGCTTTGATTCTCAG/CTGCAGGCAACCTTTAGGCGAC	
*yebE*terf/*yebE*terr	GT**GGTACC**CTGTGATTGTCACTAGCG/CATC**CCATGG**CTAGCGTTTCAAGCTCG	
For cDNA synthesis and RT-qPCR amplification		
LlBglSaF/LlBglSaR	GCATGGAATCCAGTTGACGG/GCAATTCTCAAGCCTTCAGGG	
LlCcpAaF/LlCcpAaR	AAAAGACGCGCCAGAAGGTC/TGGAATCGATATCATCAACCCC	
LlCelBaF/LlCelBaR	GGAGTCAATGACCTCGCTGG/GGTTTCCAAGCGGCAAGTC	
LlPtcAaF/LlPtcAaR	TTATCATGAGTGGAGGAAATGCC/TTTTTCGCCTTGAGCTAAACG	
LlPtcBbF/LlPtcBbR	ACAGCGGATATTGATAACATGCTTG/ATCTCCGCGCATCATTCC	
LlPurMaF/LlPurMaR	ATTGCGTAGCCATGTGCGTC/CTGTTTCTCCACCAATCAGCG	
LlTufaF/LlTufaR	CGTGACCTCTTGAGCGAATACG/GAGTGGTTTGTCAGTGTCGCG	
LlYbhEaF/LlYbhEaR	CAGCAACATTTGGTCCTTGGC/TGCTTGGCTCATCGCTTTAAG	

^a^ Strains obtained in this study are deposited in the publicly accessible IBB PAS laboratory culture collection

^b^ Amp, ampillicin; Em, erythromycin; Km, kanamycin; r, resistance; s, sensitivity

^c^ INRA. Institut National de la Recherche Agronomique (Jouy-en-Josas. France)

^d^ All primers were designed on the basis of the *L. lactis* IL1403 genome nucleotide sequence (Bolotin et al. 2001), which is available from NCBI (http://www.ncbi.nlm.nih.gov/genome) with accession no. AE005176. To certain primers restriction sites were added for digestion with ***Eco*** **RI**, ***Kpn*** **I**, ***Nco*** **I**, ***Pst*** **I** and ***Sal*** **I**

### Construction of the *claR* deletion mutants and complementing plasmid

The mutant was created by double crossover between pGhost9 harboring DNA fragments overlapping the *claR* gene and the chromosomal region containing these DNA fragments. The overlapping DNA fragments were amplified using the appropriate forward and reverse primer pairs (Table 1). The *claR* upstream region was amplified with the primers *claR*PstI/*claR*ERpr (Table 1) using ExTaq polymerase and was cloned as a *Pst*I/*Eco*RI fragment into the corresponding sites in pGhost9. The *claR* downstream region was amplified using the primers *claR*ERrv/*claR*SalI (Table 1) and was cloned as an *Eco*RI/*Sal*I fragment into the vector carrying the upstream fragment, resulting in the pGhost9Δ*claR* plasmid. The deletion plasmid was introduced into *L. lactis* IL1403 and IL1403Δ*ccpA*. Homologous recombination was enforced by 10^−3^ dilution in fresh G-M17-Em medium of the overnight culture of the lactococcal strains harboring pGhost9Δ*claR*. Diluted cultures were incubated for 2.5 h in G-M17 at non-permissive temperature (38 °C). Integrants containing pGhost9Δ*claR* in the chromosome were selected at 38 °C on G-M17 agar plates containing erythromycin. Excision from the chromosome and removing of the integration vector from *L. lactis* were performed by growth of integrants in the absence of antibiotic for at least 100 generations at the permissive temperature of 28 °C. The genetic structure of the resulting *claR* deletion strains, *L. lactis* IL1403Δ*claR*, was confirmed by colony PCR, determination of the strain sensitivity to Em, and sequencing of the DNA region containing the deleted *claR* gene.

In order to complement the *claR* deletion, the *claR* gene with its putative promoter region was amplified using *claR*Smaf and *claR*PstR primers (Table 1), cloned into pGEM-T, and transferred into *E. coli* TG1. The resultant plasmid DNA was isolated, digested with *Pst*I and *Sma*I, ligated to pIL253, digested with the same restriction enzymes, and transferred into *L. lactis* IL1403Δ*claR* giving rise to the IL1403Δ*claR*pIL253*claR* strain.

### Cloning and activity measurement of the putative intrinsic terminator following the *yebE* gene

A DNA sequence resembling a rho-independent terminator downstream of *yebE* was amplified using *yebE*terf and *yebE*terr primers (Table 1). Further cloning procedures into pGBT58 bearing the *xylE* gene that encodes catechol 2,3-dioxygenase (Table 1) and measurements of its expression determined by enzymatic assay were performed as described previously (Aleksandrzak-Piekarczyk et al. 2011). Its activity was determined in three independent experiments. As a reference point, measurements of catechol 2,3-dioxygenase activity were also performed in the wild-type pGBT58 vector, which contains no terminator located between the *xylE* gene and its promoter.

### Cloning of putative *claR* promoter region upstream of the *luxAB* genes

Construct consisting of pGEM-T, pJIM2374, and a non-coding region (amplified with *yebE*terf and *yebE*terr), containing a putative promoter of *claR* controlling the *luxAB* genes, was prepared as described previously (Aleksandrzak-Piekarczyk et al. 2011). Since pGEM-T is non-replicative in Gram-positive bacteria and pJIM2374 does not contain the *rep* gene, the recombinant pJIM2374*claRp* plasmid was transferred into *L. lactis* LL302, which encodes the RepA protein in its chromosome. The existence of a functional promoter in the cloned region was concluded from luciferase activity measurements performed in C-CDM.

### Relative quantification of the gene expression by real-time quantitative PCR

For real-time quantitative PCR (RT-qPCR), total RNA was isolated with the use of TRI Reagent (Sigma) according to the instructions of the manufacturer, from 25 ml of the IL1403 wild-type strain and its mutants (IL1403Δ*ccpA* and IL1403Δ*claR*) cultures harvested in mid-exponential phase (OD_600_ = 0.6). Cells were grown in G-CDM, C-CDM, Gal-CDM, A-CDM, or GalC-CDM. RNA was extracted from at least three independent cultures.

First-strand cDNA was synthesized from DNAse I (Sigma)-treated 2 μg RNA samples. The synthesis of cDNA was performed by the use of the High-Capacity cDNA Reverse Transcription kit (Applied Biosystems) according to manufacturer’s instructions.

Real-time quantitative PCR assays were carried out on the 7,500 Real Time PCR System (Applied Biosystems). Each reaction was carried out in reaction mixture containing the following: 1× concentrated commercial buffer supplied with polymerase (Metabion), 0.625 U Taq polymerase (Metabion), 4 mM MgCl_2_, 30,000× diluted SYBR Green (Sigma), 5 % DMSO, 0.5 ng/μl acetylated BSA (Sigma), 0.8 % glycerol (ROTH), 400 μM dNTP mixture (Metabion), forward and reverse specific primers (400 μM each), cDNA template, and water up to 25 μl of final volume. Reactions were performed with an initial denaturation step (95 °C for 3 min) followed by 45 cycles of denaturation (95 °C for 15 s) and primer annealing-extension (60 °C for 1 min). Fluorescence was read during the annealing-extension step of each cycle. After cycling, melting-point temperature analysis was performed in the range of 60 to 95 °C with temperature increments of 0.33 °C. In each experiment, background range was adjusted automatically and the threshold for C_t_ evaluation was adjusted manually. For each cDNA sample, six reactions were carried out using three template amounts, each in duplicate. For each gene studied, the amounts of cDNA were chosen individually (if possible the same for all genes) to obtain C_t_ values in range between 14 and 34 cycles. Quality of results was evaluated based on expected C_t_ differences among three cDNA amounts as well as product melting curves. Rare outlying results were omitted in calculations. The results were normalized by the use of *L. lactis*, the reference *tuf* and *purM* genes, which code for elongation factor TU and phosphoribosylaminoimidazole synthetase, respectively. Specific primers for each gene (Table 1) were designed using Primer Express software (Applied Biosystems). Before use, the primers were verified for equal efficiency of PCR reaction. The resulting data were processed using files exported from the qPCR cycler program and imported into Excel sheets to facilitate calculation of expression ratios between target and reference genes. These ratios were calculated by ΔC_t_ method using geometric mean of reference genes C_t_s in each experiment separately.

### Phenotypic testing of carbon source utilization

The study of phenotypic changes in mutant cells compared to parental strains was carried out through growth tests and analysis of fermentation patterns.

Growth tests were performed on a Microbiology Reader Analyser, Bioscreen C (Labsystems) in 0.2 ml of CDM supplemented with the required sugars. Changes in the OD_600_ of the bacterial cultures were recorded every 30 min of growth up to 100 h.

The fermentation patterns of 49 sugars were determined using the API 50CH test as specified by the manufacturer (BioMérieux) and recorded after 12, 24, and 36 h of incubation under aerobic or anaerobic conditions at 30 °C.

Further metabolic profiles were measured globally by the Phenotype MicroArrays system (Biolog, USA) according to manufacturer’s instructions. *L. lactis* strains were streaked on plates containing G-M17 agar. Colonies were scraped from the plates and titrated into IF-0a inoculating fluid (Biolog) with growth supplements and Biolog redox tetrazolium dye until the solution reached desired transmittance, according to standard protocols recommended by Biolog for *Streptococcus* species. One hundred microliters of aliquots was added to each well of carbon source plates (PM1 and PM2). The plates were incubated at 30 °C in an aerobic OmniLog incubator plate reader, and the metabolic activity was measured kinetically by determining the colorimetric reduction of a tetrazolium dye. Phenotype MicroArrays use Biolog’s redox assays, engaging cell respiration or fermentation as a universal reporter and provide precise quantitation of phenotypes. If the phenotype is strongly “positive” in a well, the cells are metabolically active, reducing a tetrazolium dye and forming a strong color. If their metabolic activity is slowed or stopped, less color or no color is formed. Data were collected approximately every 10 min over a 72-h period. This was a sufficient time for color development in the positive control wells, while the negative control wells remained colorless. Data were analyzed with the Biolog Kinetic and Parametric software. The PM1 and PM2 Biolog assays assess the ability of a bacterium to utilize any of 190 carbon compounds used in the assay as the sole carbon source.

## Results

### Structural characterization of DNA region encompassing the *claR* gene

**Fig. 1:**
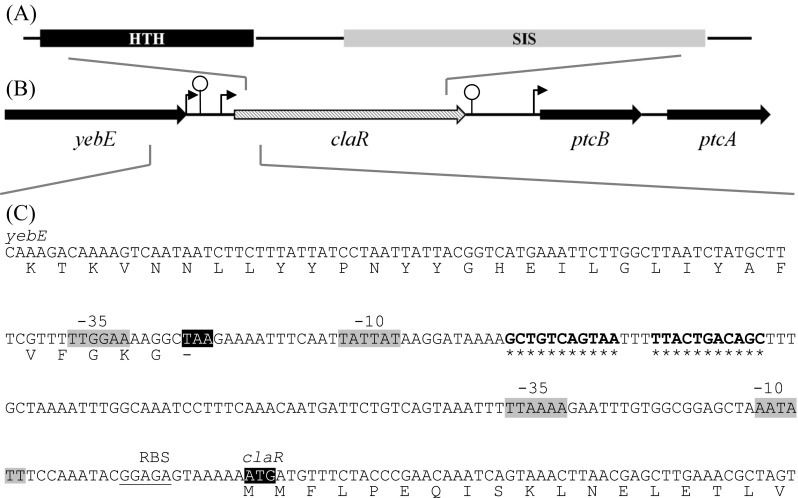
The organization of ClaR domains (**a**), the *claR* chromosomal region (**b**), and nucleotide sequence of the *yebE-claR* intergenic region (**c**). The ClaR domains (**a**) and the genes of the *claR* chromosomal region (**b**) are drawn to scale. Stem-loop structures denote rho-independent terminators. *Gray-shaded sequences* (**c**) highlight the potential −10/−35 promoter regions upstream of *claR*. The putative ribosome binding site (*RBS*) is *underlined*, and the start and stop codons of *claR* and *yebE*, respectively, are shown as *black shading. Stars* following *yebE* indicate a rho-independent terminator, the functionality of which was measured in this work

Figure 1 depicts the chromosomal region of the *claR* gene (formerly *yebF*) from *L. lactis* IL1403. ClaR reveals high-sequence similarity with transcriptional regulators of the RpiR family: it is a two-domain protein, with a 67-residue N-terminal DNA binding helix-turn-helix (HTH) motif and a 123-residue C-terminal sugar isomerase (SIS) domain (Fig. 1a; http://pfam.sanger.ac.uk/).

Located upstream of *claR* is the *yebE* gene, which encodes a hypothetical protein. Within the intergenic region between these two genes, a potential rho-independent terminator with the free energy value (dG) of −15 kcal/mol was localized (Fig. 1b, c). In order to verify its functionality, the terminator was cloned into the pGBT58 vector between a promoter and the *xylE* gene encoding a catechol 2,3-dioxygenase. In an *E. coli* strain harboring this construct levels of catechol, 2,3-dioxygenase activity obtained reached 0.50 ± 0.04 U, lower than the observed control value (pGBT58 without *yebE* terminator) of 1.25 ± 0.16 U. Thus, the presence of the *yebE* terminator caused a 60 % reduction in catechol oxygenase activity when compared to the strain harboring the wild-type pGBT58 vector.

According to an in silico analysis, the *claR* gene is preceded by at least two putative promoters (Fig. 1b, c) suggesting multiple potential transcription start sites. None of the identified potential −10 and −35 promoter regions upstream of *claR* had a full agreement with promoter consensus sequences, defined as TATAAT and TTGACA, respectively (Browning and Busby 2004). One of these potential promoters was localized upstream of the *yebE* rho-independent terminator (Fig. 1b, c). In order to verify whether in the DNA region upstream of *claR* a functional promoter (s) may exist, a DNA fragment encompassing the abovementioned putative promoters was inserted in front of the *luxAB* reporter genes in the promoter probe pJIM2374 vector and introduced into *L. lactis* LL302. Considerable luciferase activity was detected when the *claR* upstream region was tested indicating functionality of at least one promoter embedded there.

### Phenotypes of *claR* deletion mutants

**Fig. 2:**
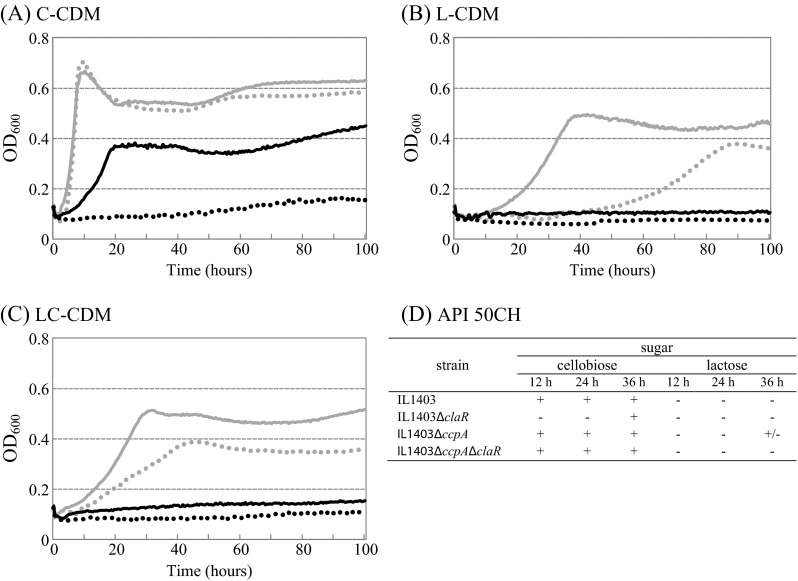
Growth of the *L. lactis* IL1403 wild-type strain (*gray dots*) and its derivatives: IL1403Δ*ccpA* (*gray line*), IL1403Δ*claR* (*black dots*), and IL1403Δ*ccpA*Δ*claR* (*black line*) in CDM containing cellobiose (**a**), lactose (**b**), or lactose + cellobiose (**c**). Sugar concentrations were 1 % except for 0.01 % cellobiose in lactose + cellobiose-supplemented CDM. *x*-axis = time (in hours), and the *y*-axis = optical density at 600 nm. Sugar fermentation patterns of the *L. lactis* IL1403 wild-type strain and its derivatives: IL1403Δ*ccpA*, IL1403Δ*claR*, and IL1403Δ*ccpA*Δ*claR* determined by the API 50CH test (**d**). The results are shown for 12, 24, and 36 h of incubation; +, good fermentation; +/−, weak fermentation; −, no fermentation (**d**)

In our previous study, the lactose-fermenting *ccpA* mutant was randomly mutagenized using pGhost9::IS*S1*. By the use of this procedure, on X-Gal supplemented plates, we have isolated several β-galactosidase-negative phenotype mutants in the *ccpA* mutant background. Among them, the inactivation of *claR* (*yebF*) in the *ccpA* mutant led to the loss of its lactose fermentation ability (Aleksandrzak-Piekarczyk et al. 2005). Thus, in this study, deletion of *claR* was performed both in the IL1403 wild-type strain and IL1403Δ*ccpA* resulting in IL1403Δ*claR* and IL1403Δ*ccpA*Δ*claR* mutants, respectively. Subsequently, IL1403Δ*claR* and IL1403Δ*ccpA*Δ*claR* growth was tested in CDM supplemented with various sugars and compared with their respective parental strains. In C-CDM, the IL1403Δ*claR* mutant initially showed no growth ability, and a minor increase in optical density of cells was observed after prolonged incubation (Fig. 2a). Deletion of *ccpA* in the IL1403Δ*claR* strain led to partial growth restoration of the obtained IL1403Δ*ccpA*Δ*claR* strain in C-CDM (Fig. 2a). Both IL1403Δ*claR* and IL1403Δ*ccpA*Δ*claR* displayed no growth in l-CDM in comparison to their parental IL1403 and IL1403Δ*ccpA* strains (Fig. 2b). The supplementation of this medium with an inducing concentration of cellobiose (LC-CDM) did not restore mutants’ growth (Fig. 2c). Deletion of the *claR* gene in the IL1403 and IL1403Δ*ccpA* strains had no effect on mutants’ growth in arbutin-, esculin-, galactose-, glucose-, or salicin-supplemented medium (data not shown).

After a 24 h incubation period, the API 50CH test confirmed the complete inability of IL1403Δ*claR* to ferment lactose and cellobiose. While the inability to assimilate lactose by both IL1403Δ*claR* and IL1403Δ*ccpA*Δ*claR* mutants lasted for 36 h, efficient utilization of cellobiose was observed in IL1403Δ*claR* after the initial 24 h (Fig. 2d). When assessed by API 50CH, no differences were found between the IL1403Δ*ccpA*Δ*claR* and its parental IL1403Δ*ccpA* within the context of cellobiose fermentation (Fig. 2d).

Global measurements of metabolic activity using the Phenotypic MicroArray approach indicated that deletion of *claR* led to complete abolishment of lactose utilization in IL1403Δ*claR*. In contrast, when cellobiose was available as a sole carbon source, this caused only a slight decrease of IL1403Δ*claR* metabolic activity compared to wild type (data not shown).

No further pronounced differential phenotypes could be identified between strains tested on all other 47 sugars available on API 50CH or the 188 carbon sources available on Biolog PM1 and PM2 arrays.

Transformation of pIL253*claR* into IL1403Δ*claR*, which led to the creation of the IL1403Δ*claR*pIL253*claR* strain, fully complemented the effects of *claR* deletion, restoring the mutant’s growth in cellobiose- and/or lactose-containing medium (data not shown).

### ClaR is expressed constitutively at low level and independently from CcpA-mediated regulation

Apart from direct regulation of gene expression mediated by CcpA binding to *cre* sequences (Kowalczyk et al. 2007), indirect control has been observed previously (Ludwig et al. 2002). Although the *claR* upstream DNA region lacks a sequence resembling the *cre* box, its expression could be still regulated by CcpA in an indirect manner. To test whether this phenomenon also occurs in the case of regulation of *claR* and whether its expression is influenced by the presence of different sugars, levels of *claR* expression were estimated by real-time quantitative RT-PCR in response to arbutin, cellobiose, galactose, and glucose in IL1403 and IL1403Δ*ccpA*. In both of these strains and in presence of all sugars tested, the *claR* gene was constitutively expressed at a low level reaching an approximate mean relative gene expression level value of 0.05 (data not shown). This suggests that *claR* expression is neither induced nor repressed by any of the sugars tested and does not undergo CcpA-dependent regulation.

### Expression of *celB*, *bglS*, and *ptcBA* genes is positively regulated by ClaR in the presence of cellobiose

Previously, we showed that genes encoding PTS^Cel-Lac^ components (*celB* and *ptcBA*) and *bglS* are negatively regulated by CcpA in *L. lactis* IL1403 (Aleksandrzak-Piekarczyk et al. 2011). In order to estimate the influence of ClaR on the expression of *bglS*, *celB*, and *ptcBA* in response to the presence of different carbon sources, RNAs were extracted from independent bacterial cultures of IL1403 and IL1403Δ*claR* grown in C-CDM, G-CDM, Gal-CDM, or GalC-CDM. Galactose in GalC-CDM was used to support the growth of IL1403Δ*claR*, since this mutant is unable to utilize cellobiose as a carbon source. Lactose was not included in the array of sugars due to *L. lactis* IL1403 poor growth parameters (growth rate, lag phase, and optical density of the final culture) in comparison with other sugars used (such as cellobiose, galactose, or glucose).

The striking difference between the levels of *bglS* and *celB* expression in IL1403 wild-type and IL1403Δ*claR* was evident when the strains were grown in GalC-CDM. In this case, levels of *celB* and *bglS* expression in the presence of ClaR (in IL1403) were significantly higher than the values obtained in the absence of ClaR (in IL1403Δ*claR*) reaching ClaR activation ratio (calculated as a quotient of relative gene expression in IL1403 and IL1403Δ*claR*) of 8 and 36, respectively (Table 2). In the case of *ptcBA*, ClaR activation ratios were close to 3, suggesting minor ClaR-dependent regulation (Table 2).

**Table 2:** ClaR and cellobiose activation ratios and the relative gene expression levels in IL1403 wild-type and IL1403Δ*claR* measured by RT-qPCR in response to different sugars

Gene	Relative gene expression level											
	Galactose		Glucose		Cellobiose/galactose		Cellobiose	ClaR activation ratio^a^ on			Cellobiose activation ratio^b^ in	
	IL1403	IL1403Δ*claR*	IL1403	IL1403Δ*claR*	IL1403	IL1403Δ*claR*	IL1403	Glucose	Galactose	Galactose/cellobiose	IL1403	IL1403 Δ*claR*
*bglS*	0.069 ± 0.019	0.070 ± 0.018	0.009 ± 0.002	0.008 ± 0.002	1.743 ± 0.485	0.048 ± 0.013	4.206 ± 0.971	1.1	1.0	36.1	25.2	0.7
*celB*	0.066 ± 0.016	0.070 ± 0.013	0.010 ± 0.002	0.005 ± 0.001	0.487 ± 0.128	0.058 ± 0.014	2.040 ± 0.438	1.8	1.0	8.4	7.3	0.8
*ptcA*	1.532 ± 0.165	0.952 ± 0.220	0.030 ± 0.007	0.029 ± 0.004	5.907 ± 1.011	1.515 ± 0.105	17.215 ± 0.490	1.0	1.6	3.9	3.9	1.6
*ptcB*	7.536 ± 1.509	8.391 ± 1.927	0.177 ± 0.05	0.204 ± 0.057	24.006 ± 6.003	9.773 ± 2.118	36.166 ± 6.054	0.9	0.9	2.5	3.2	1.2
*claR*	0.046 ± 0.008	–	0.061 ± 0.010	–	0.049 ± 0.012	–	0.037 ± 0.009	–	–	–	1.1	–

(–) not determined

^a^ClaR activation ratio was calculated as a quotient of relative gene expression in IL1403 and IL1403Δ*claR*

^b^Cellobiose activation ratio was calculated as a quotient of relative gene expression in the wild-type strain IL1403 or IL1403Δ*claR* grown in GalC-CDM and in Gal-CDM

The lowest transcript levels were detected in IL1403 wild-type and IL1403Δ*claR* cells growing under repressive conditions (G-CDM) due to probable downregulation of *bglS*, *celB*, and *ptcBA* expression by CcpA. Levels of *bglS* and *celB* expression were only slightly varied, whereas the *ptcBA* transcription was increased in both strains in medium supplemented with galactose (Table 2). Comparable expression levels between IL1403 wild-type and IL1403Δ*claR* strains in the presence of galactose or glucose (ClaR activation ratio ∼1; Table 2) imply that these sugars do not elicit ClaR-dependent activation of *bglS*, *celB*, and *ptcBA*.

Substantially higher transcription levels of all genes tested were detected when the wild-type strain was grown in a medium supplemented with cellobiose (C-CDM) as a sole carbon source (Table 2). The addition of galactose (GalC-CDM) caused a 2–4-fold transcription decrease of *bglS*, *celB*, and *ptcAB* when compared to C-CDM. This may suggest that the presence of galactose leads to the repression of transcription in a similar manner to glucose.

Furthermore, to determine the influence of cellobiose on *celB*, *bglS*, *ptcB*, and *ptcA* transcription in the presence (in IL1403) or absence (in IL1403Δ*claR*) of ClaR cellobiose, activation ratios were calculated as a quotient of relative gene expression in the wild-type strain IL1403 or IL1403Δ*claR* grown in GalC-CDM and in Gal-CDM. These ratios obtained in IL1403 varied from 25 (*bglS*) and 7 (*celB*) to as low as 3–4-fold (*ptcBA*) implying that cellobiose has different levels of influence on the activation of associated genes (Table 2). These cellobiose activation ratios were similar to ClaR activation values calculated for GalC-CDM suggesting that this cellobiose-dependent regulation may be due to ClaR action. This was confirmed by the observation that the cellobiose activation ratios obtained in the absence of ClaR were generally around a factor of 1, indicating a lack of genetic expression regulation in IL1403Δ*claR* even in the presence of cellobiose (Table 2).

## Discussion

The *claR* gene product from *L. lactis* IL1403 belongs to the RpiR family of potential regulators, of which members are abundantly represented in many other related or distantly related bacteria from different genera (http://www.kegg.jp/kegg/ssdb/). The strongest ClaR orthologs are found only in other *Lactococcus* species (e.g., *L. lactis* subspecies *cremori*s MG1363 and SK11, and *L. lactis* subspecies *lactis* KF147). There are eight paralogs (encoded by *yidA*, *yecA*, *yljC*, *yfeA*, *yugA*, *gntR*, and *yleF*) from the RpiR family, with ClaR being the prototypical example, encoded in the *L. lactis* IL1403 genome (Bolotin et al. 2001; Guédon et al. 2002). Thus far, no function has been assigned to any of the RpiR family genes in *L. lactis*.

However, the conservation of ClaR orthologs found in many different bacterial species suggests an important role in host organisms. Indeed, few members of the RpiR family have been shown to function as positive or negative transcriptional regulators of genes involved in the metabolism of different carbon sources. For example, in *Bacillus subtilis*, maltose metabolism is positively regulated by GlvR (Yamamoto et al. 2001). Until now, GlvR was the only representative from the family of RpiR regulators characterized within a group of Gram-positive bacteria. All other functionally characterized examples originate from Gram-negative bacteria, where RpiR family regulators appear to downregulate gene expression. These RpiR-like repressors have been shown to regulate the metabolism of ribose and *N*-acetylmuramic acid in *E. coli*, glucose in *Pseudomonas putida*, and inositol in *Caulobacter crescentus*, *Salmonella enterica*, and *Sinorhizobium meliloti* (Boutte et al. 2008; Daddaoua et al. 2009; Jaeger and Mayer 2008; Kohler et al. 2011; Kröger and Fuchs 2009; Sørensen and Hove-Jensen 1996). Members of the RpiR family contain a HTH motif at their N-terminus and a SIS domain on their C-terminal end. The SIS domain is a phospho-sugar-binding domain found in many proteins that regulate the expression of genes involved in synthesis of phospho-sugars (Bateman 1999; Teplyakov et al. 1998).

It is tempting to speculate that this type of regulation is operative in *L. lactis* IL1403; theoretically, phosphorylated upon entry into the IL1403 cell via CelB cellobiose or lactose bind to the SIS domain of ClaR. Such association may result in a stimulation of the ClaR regulator and allows its binding to the respective DNA regions, resulting in ClaR-dependent regulation of gene expression. We postulate that through this mechanism the expression of *bglS*, *celB*, and *ptcBA* could be activated in the presence of cellobiose or lactose. Evidence confirming this assumption includes strong observed decreases in *bglS*, *celB*, and, to a lesser extent, *ptcBA* mRNA levels in response to ClaR absence upon the growth of a *claR* mutant in cellobiose-containing medium (Table 2). This effect is not observable when galactose or glucose is present as a sole carbon source, indicating that these sugars have no activating role toward ClaR.

Additionally, the hypothesis that ClaR plays a role in lactose and cellobiose metabolism is strongly confirmed by the growth deficiencies of *claR* mutants in cellobiose- and/or lactose-supplemented media (Fig. 2a–c). Deletion of *ccpA* led to the partial growth restoration of the IL1403Δ*ccpA*Δ*claR* mutant in cellobiose-supplemented media, but not in lactose-supplemented media (Fig. 2a–c). Thus, it seems reasonable to conclude that both mutants are barely or not able to utilize these sugars due to a lack of observed *bglS* and *celB* transcriptional activation by ClaR. Indeed, in the absence of ClaR, transcription of *bglS* and *celB* decreases 36- and 8-fold, respectively (Table 2). This may result in a severe decrease in BglS and CelB, which are indispensable for *L. lactis* IL1403 growth on cellobiose and lactose as shown previously (Aleksandrzak-Piekarczyk et al. 2005, 2011).

When assayed globally (by Phenotype MicroArrays and API 50CH), no additional pronounced differences in utilization of tens of different carbon sources were detected between the *claR* mutants and their parental strains. This implies a narrow but significant specificity of ClaR. The contradictory phenomenon that IL1403Δ*claR* is barely able to grow on cellobiose (Fig. 2a) while efficiently respiring on this sugar (when tested by phenotype MicroArrays) could be explained by the fate of cellobiose metabolism in IL1403. Previously, we showed that the EIIC CelB permease is the only protein dedicated to cellobiose and lactose uptake in *L. lactis* IL1403 (Aleksandrzak-Piekarczyk et al. 2011), while BglS is the only glycosidase crucial for lactose hydrolysis (Aleksandrzak-Piekarczyk et al. 2005). Although cellobiose is also a substrate for BglS, it was postulated that one or more additional enzyme(s) capable of hydrolysis of this sugar may exist in IL1403 and may be subject to CcpA-mediated negative regulation (Aleksandrzak-Piekarczyk et al. 2005). Moreover, results of the current study clearly indicate that the transcription of *bglS* tightly depends on its activation by ClaR, whereas transcription of *celB* is to a lesser extent dependent on the activation by ClaR (Table 2). Thus, we may assume that in IL1403Δ*claR*, small amounts of CelB are still present and may transport traces of lactose or cellobiose. Internalized by IL1403Δ*claR*, lactose does not serve as an energy source due to a lack of BglS resulting from the absence of ClaR. On the other hand, in IL1403Δ*claR*, small amounts of cellobiose-derived metabolites, which are formed after cellobiose uptake via CelB and activity of different than BglS hydrolases, are insufficient for effective mutant cell division, resulting in the limited mutant’s growth.

The results determined during this study by RT-qPCR indicate that *claR* is weakly expressed under all conditions tested (in presence of different sugars [Table 2] and in presence or absence of the CcpA protein [data not shown]), in which it reaches the relative gene expression levels only slightly above zero. This is in agreement with the fact that many transcriptional regulators exist endogenously in low amounts, and, in the case of ClaR, it may be due to several reasons. First, both of the putative promoters found upstream of the *claR* gene show incomplete correlation with the consensus sites for both −10 and −35 sequences, and thus may drive a low level transcription. Second, one these putative promoters yield transcript that starts upstream of the rho-independent terminator sequence (Fig. 1b, c) that may prematurely terminate *claR* transcription. We proved that it is a moderately strong terminator, as it arrested the transcription of *claR* with about 60 % efficiency. Thus, it might be assumed that the generation of full-length transcript covering the *claR* gene is likely achieved by a low amount of read-through transcription. However, we cannot exclude the possibility that the efficiency of read-through termination could be strengthened by the influence of unknown antitermination factors.

Despite the narrow specificity of ClaR within the context of sugar metabolism, it seems that this regulator has an invaluable role in lactococcal cells. We demonstrated that its activity is crucial for the assimilation of two sugars that are commonly found in ecosystems inhabited by *L. lactis* strains—plant material (cellobiose) and milk (lactose). Having such a regulator allows for a settlement of more diverse environments, and thus gives *L. lactis* an advantage over other microorganisms. The unprecedented role of the ClaR protein is made even more significant by the fact that the metabolism of lactose and β-glucosides is of great economic importance for biotechnological processes involving *L. lactis*, in regard to the production of both fermented milk and plant products. Obtaining detailed knowledge related to sugar metabolism and the regulation of associated gene expression in *L. lactis* may contribute to the improvement of mechanisms controlling significant cellular processes in these bacteria. In the case of industrial microorganisms such as *L. lactis*, modification of defined regulatory networks may drastically affect the properties of the bacteria and have implications on bioprocesses. Finally, we can assume that through our research, we can introduce changes in the metabolic potential of *Lactococcus* strains, which by themselves are not able to assimilate lactose. We can initiate this process through the inactivation of *ccpA* or activation of other genes by addition of cellobiose. In contrast to plasmid-located *lac*-operons, genes encoding PTS^Cel-Lac^ components are located on the chromosome, which ensures their stability, a potentially important feature for industrial applications.

## References

[CR1] Aleksandrzak-Piekarczyk T (2013) Lactose and β-glucosides metabolism and its regulation in *Lactococcus lactis*: a review. In: Kongo JM (ed) Lactic acid bacteria—R & D for food, health and livestock purposes. InTechOpen. doi: 10.5772/50889

[CR2] Aleksandrzak-Piekarczyk T, Kok J, Renault P, Bardowski J (2005). Alternative lactose catabolic pathway in *Lactococcus lactis* IL1403. Appl Environ Microbiol.

[CR3] Aleksandrzak-Piekarczyk T, Polak J, Jezierska B, Renault P, Bardowski J (2011). Genetic characterization of the CcpA-dependent, cellobiose-specific PTS system comprising CelB, PtcB and PtcA that transports lactose in *Lactococcus lactis* IL1403. Int J Food Microbiol.

[CR4] Bardowski J, Ehrlich SD, Chopin A (1994). BglR protein, which belongs to the BglG family of transcriptional antiterminators, is involved in beta-glucoside utilization in *Lactococcus lactis*. J Bacteriol.

[CR5] Barrière C, Veiga-da-Cunha M, Pons N, Guédon E, van Hijum SAFT, Kok J, Kuipers OP, Ehrlich DS, Renault P (2005). Fructose utilization in *Lactococcus lactis* as a model for low-GC Gram-positive bacteria: its regulator, signal, and DNA-binding site. J Bacteriol.

[CR6] Bateman A (1999). The SIS domain: a phosphosugar-binding domain. Trends Biochem Sci.

[CR7] Bolotin A, Wincker P, Mauger S, Jaillon O, Malarme K, Weissenbach J, Ehrlich SD, Sorokin A (2001). The complete genome sequence of the lactic acid bacterium *Lactococcus lactis* ssp. *lactis* IL1403. Genome Res.

[CR8] Boucher I, Vadeboncoeur C, Moineau S (2003). Characterization of genes involved in the metabolism of α-galactosides by *Lactococcus raffinolactis*. Appl Environ Microbiol.

[CR9] Boutte CC, Srinivasan BS, Flannick JA (2008). Genetic and computational identification of a conserved bacterial metabolic module. PLoS Genet.

[CR10] Browning DF, Busby SJW (2004). The regulation of bacterial transcription initiation. Nat Rev Microbiol.

[CR11] Chopin A, Chopin M-C, Moillo-Batt A, Langella P (1984). Two plasmid-determined restriction and modification systems in *Streptococcus lactis*. Plasmid.

[CR12] Daddaoua A, Krell T, Ramos J-L (2009). Regulation of glucose metabolism in *Pseudomonas*: the phosphorylative branch and Entner-Doudoroff enzymes are regulated by a repressor containing a sugar isomerase domain. J Biol Chem.

[CR13] Delorme C, Ehrlich SD, Renault P (1999). Regulation of expression of the *Lactococcus lactis* histidine operon. J Bacteriol.

[CR14] Guédon E, Jamet E, Renault P (2002). Gene regulation in *Lactococcus lactis*: the gap between predicted and characterized regulators. Antonie Van Leeuwenhoek.

[CR15] Hueck CJ, Hillen W (1995). Catabolite repression in *Bacillus subtilis*: a global regulatory mechanism for the Gram-positive bacteria?. Mol Microbiol.

[CR16] Jaeger T, Mayer C (2008). The transcriptional factors MurR and catabolite activator protein regulate N-acetylmuramic acid catabolism in *Escherichia coli*. J Bacteriol.

[CR17] Jagura-Burdzy G, Khanim F, Smith CA, Thomas CM (1992). Crosstalk between plasmid vegetative replication and conjugative transfer: repression of the *trfA* operon by *trbA* of broad host range plasmid RK2. Nucleic Acids Res.

[CR18] Kohler PRA, Choong E-L, Rossbach S (2011). The RpiR-like repressor IolR regulates inositol catabolism in *Sinorhizobium meliloti*. J Bacteriol.

[CR19] Kowalczyk M, Borcz B, Płochocka D, Bardowski J (2007). In vitro DNA binding of purified CcpA protein from *Lactococcus lactis* IL1403. Acta Biochim Pol.

[CR20] Kröger C, Fuchs TM (2009). Characterization of the myo-inositol utilization island of *Salmonella enterica* serovar Typhimurium. J Bacteriol.

[CR21] Leenhouts K, Bolhuis A, Venema G, Kok J (1998). Construction of a food-grade multiple-copy integration system for *Lactococcus lactis*. Appl Microbiol Biotechnol.

[CR22] Lorca G, Reddy L, Nguyen A, Sun EI, Tseng J, Yen M-R, Saier MH, Mozzi F, Raya RR, Vignolo GM (2010). Lactic acid bacteria: comparative genomic analyses of transport systems. Biotechnology of lactic acid bacteria: novel applications.

[CR23] Ludwig H, Rebhan N, Blencke H-M, Merzbacher M, Stülke J (2002). Control of the glycolytic *gapA* operon by the catabolite control protein A in *Bacillus subtilis*: a novel mechanism of CcpA-mediated regulation. Mol Microbiol.

[CR24] Luesink EJ, Van Herpen REMA, Grossiord BP, Kuipers OP, De Vos WM (1998). Transcriptional activation of the glycolytic *las* operon and catabolite repression of the *gal* operon in *Lactococcus lactis* are mediated by the catabolite control protein CcpA. Mol Microbiol.

[CR25] Maguin E, Prévost H, Ehrlich SD, Gruss A (1996). Efficient insertional mutagenesis in lactococci and other Gram-positive bacteria. J Bacteriol.

[CR26] Mayo B, Aleksandrzak-Piekarczyk T, Fernández M, Kowalczyk M, Álvarez-Martín P, Bardowski J, Mozzi F, Raya RR, Vignolo GM (2010). Updates in the metabolism of lactic acid bacteria. Biotechnology of lactic acid bacteria: novel applications.

[CR27] Monedero V, Kuipers OP, Jamet E, Deutscher J (2001). Regulatory functions of serine-46-phosphorylated HPr in *Lactococcus lactis*. J Bacteriol.

[CR28] Simon D, Chopin A (1988). Construction of a vector plasmid family and its use for molecular cloning in *Streptococcus lactis*. Biochimie.

[CR29] Sissler M, Delorme C, Bond J, Ehrlich SD, Renault P, Francklyn C (1999). An aminoacyl-tRNA synthetase paralog with a catalytic role in histidine biosynthesis. Proc Natl Acad Sci.

[CR30] Solopova A, Bachmann H, Teusink B, Kok J, Neves AR, Kuipers OP (2012). A specific mutation in the promoter region of the silent *cel* cluster accounts for the appearance of lactose-utilizing *Lactococcus lactis* MG1363. Appl Environ Microbiol.

[CR31] Sørensen KI, Hove-Jensen B (1996). Ribose catabolism of *Escherichia coli*: characterization of the *rpiB* gene encoding ribose phosphate isomerase B and of the *rpiR* gene, which is involved in regulation of *rpiB* expression. J Bacteriol.

[CR32] Teplyakov A, Obmolova G, Badet-Denisot M-A, Badet B, Polikarpov I (1998). Involvement of the C terminus in intramolecular nitrogen channeling in glucosamine 6-phosphate synthase: evidence from a 1.6 å crystal structure of the isomerase domain. Structure.

[CR33] Terzaghi BE, Sandine WE (1975). Improved medium for lactic streptococci and their bacteriophages. Appl Microbiol.

[CR34] Weickert MJ, Chambliss GH (1990). Site-directed mutagenesis of a catabolite repression operator sequence in *Bacillus subtilis*. Proc Natl Acad Sci.

[CR35] Wood EJ (1983). Molecular cloning. A laboratory manual by T Maniatis, E F Fritsch and J Sambrook. Cold Spring Harbor Laboratory, New York. Biochem Educ.

[CR36] Yamamoto H, Serizawa M, Thompson J, Sekiguchi J (2001). Regulation of the *glv* operon in *Bacillus subtilis*: YfiA (GlvR) is a positive regulator of the operon that is repressed through CcpA and *cre*. J Bacteriol.

